# Supplementary material for: Trend of cancer mortality of the female reproductive system in China from 2005 to 2018 and prediction to 2035: A log-linear regression and Bayesian age-period- cohort analysis
Source: PLoS One. 2025 Aug 11;20(8):e0328869. doi: 10.1371/journal.pone.0328869 (PMC12338781; doi:10.1371/journal.pone.0328869)
Supplement: S1 File — (DOC) [file pone.0328869.s001.doc]

| Table S1 Mortality rate of vulvar cancer in China from 2005 to 2018 (1/100,000) | | | | | | | | | |
| --- | --- | --- | --- | --- | --- | --- | --- | --- | --- |
| Year | Nationwide | | | Urban | | | Rural | | |
|  | Number | Rate | ASMR | Number | Rate | ASMR | Number | Rate | ASMR |
| 2005 | 35 | 0.13 | 0.10 | 32 | 0.16 | 0.12 | 3 | 0.04 | 0.05 |
| 2006 | 38 | 0.13 | 0.10 | 32 | 0.14 | 0.11 | 6 | 0.09 | 0.08 |
| 2007 | 59 | 0.20 | 0.16 | 51 | 0.23 | 0.18 | 8 | 0.11 | 0.10 |
| 2008 | 59 | 0.20 | 0.16 | 51 | 0.23 | 0.18 | 8 | 0.11 | 0.10 |
| 2009 | 46 | 0.11 | 0.08 | 37 | 0.13 | 0.09 | 8 | 0.06 | 0.05 |
| 2010 | 82 | 0.13 | 0.10 | 73 | 0.18 | 0.14 | 9 | 0.04 | 0.04 |
| 2011 | 122 | 0.17 | 0.13 | 85 | 0.19 | 0.15 | 37 | 0.13 | 0.11 |
| 2012 | 141 | 0.14 | 0.11 | 92 | 0.18 | 0.13 | 49 | 0.10 | 0.09 |
| 2013 | 173 | 0.15 | 0.11 | 110 | 0.20 | 0.13 | 63 | 0.11 | 0.10 |
| 2014 | 222 | 0.16 | 0.12 | 136 | 0.19 | 0.14 | 87 | 0.12 | 0.10 |
| 2015 | 265 | 0.17 | 0.12 | 152 | 0.20 | 0.13 | 114 | 0.14 | 0.11 |
| 2016 | 312 | 0.17 | 0.12 | 190 | 0.20 | 0.14 | 122 | 0.13 | 0.10 |
| 2017 | 384 | 0.18 | 0.13 | 199 | 0.19 | 0.12 | 186 | 0.17 | 0.13 |
| 2018 | 536 | 0.21 | 0.15 | 284 | 0.24 | 0.16 | 250 | 0.18 | 0.13 |
| Total | 2210 | 0.16 | 0.12 | 1523 | 0.19 | 0.14 | 951 | 0.11 | 0.09 |

| Table S2 Mortality rate of vaginal cancer in China from 2005 to 2018 (1/100,000) | | | | | | | | | |
| --- | --- | --- | --- | --- | --- | --- | --- | --- | --- |
| Year | Nationwide | | | Urban | | | Rural | | |
|  | Number | Rate | ASMR | Number | Rate | ASMR | Number | Rate | ASMR |
| 2005 | 21 | 0.08 | 0.07 | 18 | 0.09 | 0.07 | 3 | 0.04 | 0.04 |
| 2006 | 22 | 0.07 | 0.07 | 16 | 0.07 | 0.06 | 6 | 0.09 | 0.09 |
| 2007 | 17 | 0.06 | 0.05 | 13 | 0.06 | 0.05 | 4 | 0.05 | 0.06 |
| 2008 | 17 | 0.06 | 0.05 | 13 | 0.06 | 0.05 | 4 | 0.05 | 0.06 |
| 2009 | 33 | 0.08 | 0.06 | 30 | 0.11 | 0.09 | 3 | 0.02 | 0.02 |
| 2010 | 39 | 0.06 | 0.05 | 30 | 0.08 | 0.06 | 8 | 0.04 | 0.03 |
| 2011 | 45 | 0.06 | 0.05 | 30 | 0.07 | 0.05 | 14 | 0.05 | 0.04 |
| 2012 | 84 | 0.09 | 0.07 | 47 | 0.09 | 0.07 | 35 | 0.07 | 0.06 |
| 2013 | 97 | 0.09 | 0.07 | 51 | 0.09 | 0.07 | 47 | 0.08 | 0.07 |
| 2014 | 150 | 0.11 | 0.08 | 88 | 0.12 | 0.09 | 62 | 0.09 | 0.07 |
| 2015 | 137 | 0.09 | 0.07 | 85 | 0.11 | 0.08 | 52 | 0.06 | 0.05 |
| 2016 | 186 | 0.10 | 0.07 | 120 | 0.13 | 0.09 | 65 | 0.07 | 0.06 |
| 2017 | 226 | 0.10 | 0.08 | 121 | 0.11 | 0.08 | 103 | 0.09 | 0.07 |
| 2018 | 272 | 0.11 | 0.08 | 139 | 0.12 | 0.08 | 129 | 0.09 | 0.07 |
| Total | 1208 | 0.08 | 0.07 | 802 | 0.09 | 0.07 | 536 | 0.07 | 0.06 |

| Table S3 Mortality rate of cervical uteri cancer in China from 2005 to 2018 (1/100,000) | | | | | | | | | |
| --- | --- | --- | --- | --- | --- | --- | --- | --- | --- |
| Year | Nationwide | | | Urban | | | Rural | | |
|  | Number | Rate | ASMR | Number | Rate | ASMR | Number | Rate | ASMR |
| 2005 | 654 | 2.41 | 2.11 | 428 | 2.14 | 1.78 | 226 | 3.18 | 3.22 |
| 2006 | 747 | 2.53 | 2.24 | 507 | 2.20 | 1.86 | 240 | 3.70 | 3.85 |
| 2007 | 846 | 2.86 | 2.52 | 532 | 2.41 | 2.06 | 314 | 4.17 | 4.20 |
| 2008 | 846 | 2.86 | 2.52 | 532 | 2.41 | 2.06 | 314 | 4.17 | 4.20 |
| 2009 | 1388 | 3.29 | 2.84 | 917 | 3.22 | 2.66 | 470 | 3.42 | 3.22 |
| 2010 | 2187 | 3.55 | 3.10 | 1387 | 3.50 | 2.97 | 799 | 3.65 | 3.34 |
| 2011 | 2673 | 3.71 | 3.22 | 1531 | 3.51 | 2.94 | 1142 | 4.00 | 3.70 |
| 2012 | 3744 | 3.83 | 3.29 | 1829 | 3.67 | 3.03 | 1916 | 4.01 | 3.61 |
| 2013 | 4605 | 4.12 | 3.48 | 2229 | 4.02 | 3.25 | 2375 | 4.23 | 3.73 |
| 2014 | 6709 | 4.72 | 3.94 | 3238 | 4.52 | 3.66 | 3471 | 4.93 | 4.27 |
| 2015 | 8027 | 5.08 | 4.15 | 3785 | 4.94 | 3.93 | 4241 | 5.21 | 4.38 |
| 2016 | 10270 | 5.46 | 4.43 | 5116 | 5.34 | 4.27 | 5155 | 5.59 | 4.61 |
| 2017 | 11935 | 5.55 | 4.44 | 5455 | 5.13 | 4.04 | 6480 | 5.95 | 4.85 |
| 2018 | 14737 | 5.72 | 4.55 | 6318 | 5.37 | 4.22 | 8420 | 6.01 | 4.84 |
| Total | 61345 | 3.98 | 3.34 | 33803 | 3.74 | 3.05 | 35563 | 4.44 | 4.00 |

| Table S4 Mortality rate of corpus uteri cancer in China from 2005 to 2018 (1/100,000) | | | | | | | | | |
| --- | --- | --- | --- | --- | --- | --- | --- | --- | --- |
| Year | Nationwide | | | Urban | | | Rural | | |
|  | Number | Rate | ASMR | Number | Rate | ASMR | Number | Rate | ASMR |
| 2005 | 352 | 1.30 | 1.16 | 248 | 1.24 | 1.06 | 104 | 1.46 | 1.47 |
| 2006 | 325 | 1.10 | 0.94 | 253 | 1.10 | 0.89 | 72 | 1.11 | 1.17 |
| 2007 | 412 | 1.39 | 1.19 | 293 | 1.33 | 1.06 | 119 | 1.58 | 1.63 |
| 2008 | 412 | 1.39 | 1.19 | 293 | 1.33 | 1.06 | 119 | 1.58 | 1.63 |
| 2009 | 642 | 1.52 | 1.26 | 465 | 1.63 | 1.29 | 177 | 1.29 | 1.19 |
| 2010 | 959 | 1.56 | 1.31 | 668 | 1.68 | 1.35 | 291 | 1.33 | 1.21 |
| 2011 | 1264 | 1.75 | 1.47 | 804 | 1.84 | 1.46 | 460 | 1.61 | 1.48 |
| 2012 | 1717 | 1.76 | 1.46 | 937 | 1.88 | 1.46 | 780 | 1.63 | 1.46 |
| 2013 | 2041 | 1.83 | 1.49 | 1069 | 1.93 | 1.46 | 973 | 1.73 | 1.51 |
| 2014 | 2229 | 1.57 | 1.25 | 1203 | 1.68 | 1.26 | 1027 | 1.46 | 1.24 |
| 2015 | 2496 | 1.58 | 1.24 | 1342 | 1.75 | 1.31 | 1154 | 1.42 | 1.17 |
| 2016 | 3080 | 1.64 | 1.28 | 1679 | 1.75 | 1.31 | 1398 | 1.52 | 1.23 |
| 2017 | 3452 | 1.60 | 1.24 | 1831 | 1.72 | 1.29 | 1623 | 1.49 | 1.19 |
| 2018 | 4760 | 1.85 | 1.40 | 2263 | 1.93 | 1.41 | 2497 | 1.78 | 1.39 |
| Total | 21645 | 1.56 | 1.28 | 13348 | 1.63 | 1.26 | 10794 | 1.50 | 1.36 |

| Table S5 Mortality rate of uterine cancer in China from 2005 to 2018 (1/100,000) | | | | | | | | | |
| --- | --- | --- | --- | --- | --- | --- | --- | --- | --- |
| Year | Nationwide | | | Urban | | | Rural | | |
|  | Number | Rate | ASMR | Number | Rate | ASMR | Number | Rate | ASMR |
| 2005 | 410 | 1.51 | 1.32 | 307 | 1.54 | 1.28 | 103 | 1.45 | 1.45 |
| 2006 | 461 | 1.56 | 1.36 | 345 | 1.50 | 1.24 | 116 | 1.79 | 1.86 |
| 2007 | 415 | 1.40 | 1.19 | 306 | 1.39 | 1.11 | 109 | 1.45 | 1.48 |
| 2008 | 415 | 1.40 | 1.19 | 306 | 1.39 | 1.11 | 109 | 1.45 | 1.48 |
| 2009 | 564 | 1.34 | 1.12 | 297 | 1.04 | 0.82 | 267 | 1.94 | 1.80 |
| 2010 | 788 | 1.28 | 1.08 | 446 | 1.12 | 0.91 | 342 | 1.56 | 1.41 |
| 2011 | 858 | 1.19 | 1.00 | 441 | 1.01 | 0.80 | 418 | 1.47 | 1.33 |
| 2012 | 1167 | 1.20 | 0.99 | 487 | 0.98 | 0.76 | 680 | 1.42 | 1.25 |
| 2013 | 1145 | 1.03 | 0.83 | 459 | 0.83 | 0.62 | 686 | 1.22 | 1.05 |
| 2014 | 1384 | 0.97 | 0.78 | 598 | 0.83 | 0.62 | 787 | 1.12 | 0.95 |
| 2015 | 1371 | 0.87 | 0.68 | 532 | 0.69 | 0.52 | 842 | 1.03 | 0.85 |
| 2016 | 1638 | 0.87 | 0.67 | 680 | 0.71 | 0.53 | 956 | 1.04 | 0.82 |
| 2017 | 1791 | 0.83 | 0.64 | 787 | 0.74 | 0.55 | 1004 | 0.92 | 0.73 |
| 2018 | 2037 | 0.79 | 0.60 | 825 | 0.70 | 0.51 | 1213 | 0.87 | 0.67 |
| Total | 13075 | 1.16 | 0.96 | 6815 | 1.03 | 0.81 | 7633 | 1.34 | 1.22 |

| Table S6 Mortality rate of ovarian cancer in China from 2005 to 2018 (1/100,000) | | | | | | | | | |
| --- | --- | --- | --- | --- | --- | --- | --- | --- | --- |
| Year | Nationwide | | | Urban | | | Rural | | |
|  | Number | Rate | ASMR | Number | Rate | ASMR | Number | Rate | ASMR |
| 2005 | 847 | 3.13 | 2.78 | 744 | 3.72 | 3.15 | 103 | 1.45 | 1.54 |
| 2006 | 998 | 3.38 | 2.97 | 885 | 3.84 | 3.24 | 113 | 1.74 | 1.84 |
| 2007 | 988 | 3.34 | 2.88 | 867 | 3.93 | 3.21 | 121 | 1.61 | 1.67 |
| 2008 | 988 | 3.34 | 2.88 | 867 | 3.93 | 3.21 | 121 | 1.61 | 1.67 |
| 2009 | 1454 | 3.44 | 2.92 | 1194 | 4.19 | 3.39 | 260 | 1.89 | 1.80 |
| 2010 | 2071 | 3.36 | 2.87 | 1665 | 4.20 | 3.45 | 406 | 1.85 | 1.73 |
| 2011 | 2375 | 3.29 | 2.80 | 1668 | 3.83 | 3.08 | 707 | 2.48 | 2.30 |
| 2012 | 3163 | 3.24 | 2.72 | 2009 | 4.03 | 3.19 | 1155 | 2.42 | 2.18 |
| 2013 | 3714 | 3.33 | 2.75 | 2321 | 4.18 | 3.24 | 1394 | 2.48 | 2.19 |
| 2014 | 4838 | 3.41 | 2.80 | 3014 | 4.21 | 3.28 | 1823 | 2.59 | 2.25 |
| 2015 | 5705 | 3.61 | 2.89 | 3409 | 4.45 | 3.43 | 2296 | 2.82 | 2.35 |
| 2016 | 7241 | 3.85 | 3.05 | 4415 | 4.61 | 3.54 | 2829 | 3.07 | 2.51 |
| 2017 | 7750 | 3.60 | 2.84 | 4530 | 4.26 | 3.25 | 3219 | 2.95 | 2.41 |
| 2018 | 9405 | 3.65 | 2.83 | 5098 | 4.34 | 3.26 | 4305 | 3.07 | 2.45 |
| Total | 45834 | 3.43 | 2.86 | 32685 | 4.12 | 3.28 | 18851 | 2.29 | 2.06 |

| Table S7 Mortality rate of other female genital cancers in China from 2005 to 2018 (1/100,000) | | | | | | | | | |
| --- | --- | --- | --- | --- | --- | --- | --- | --- | --- |
| Year | Nationwide | | | Urban | | | Rural | | |
|  | Number | Rate | ASMR | Number | Rate | ASMR | Number | Rate | ASMR |
| 2005 | 30 | 0.11 | 0.10 | 21 | 0.11 | 0.09 | 9 | 0.13 | 0.12 |
| 2006 | 47 | 0.16 | 0.14 | 41 | 0.18 | 0.15 | 6 | 0.09 | 0.09 |
| 2007 | 28 | 0.09 | 0.08 | 27 | 0.12 | 0.10 | 1 | 0.01 | 0.01 |
| 2008 | 28 | 0.09 | 0.08 | 27 | 0.12 | 0.10 | 1 | 0.01 | 0.01 |
| 2009 | 61 | 0.14 | 0.12 | 46 | 0.16 | 0.13 | 15 | 0.11 | 0.10 |
| 2010 | 86 | 0.14 | 0.11 | 73 | 0.18 | 0.14 | 12 | 0.06 | 0.05 |
| 2011 | 88 | 0.12 | 0.10 | 67 | 0.15 | 0.12 | 21 | 0.07 | 0.07 |
| 2012 | 196 | 0.20 | 0.17 | 127 | 0.26 | 0.20 | 69 | 0.14 | 0.13 |
| 2013 | 191 | 0.17 | 0.13 | 103 | 0.19 | 0.14 | 87 | 0.15 | 0.13 |
| 2014 | 258 | 0.18 | 0.14 | 147 | 0.21 | 0.16 | 112 | 0.16 | 0.13 |
| 2015 | 316 | 0.20 | 0.16 | 173 | 0.23 | 0.17 | 141 | 0.17 | 0.14 |
| 2016 | 368 | 0.20 | 0.15 | 228 | 0.24 | 0.18 | 140 | 0.15 | 0.12 |
| 2017 | 418 | 0.19 | 0.14 | 249 | 0.23 | 0.16 | 168 | 0.15 | 0.12 |
| 2018 | 501 | 0.19 | 0.15 | 272 | 0.23 | 0.17 | 229 | 0.16 | 0.13 |
| Total | 2300 | 0.16 | 0.13 | 1603 | 0.19 | 0.14 | 1011 | 0.11 | 0.10 |

| Table S8 Mortality rate of placenta cancer in China from 2005 to 2018 (1/100,000) | | | | | | | | | |
| --- | --- | --- | --- | --- | --- | --- | --- | --- | --- |
| Year | Nationwide | | | Urban | | | Rural | | |
|  | Number | Rate | ASMR | Number | Rate | ASMR | Number | Rate | ASMR |
| 2005 | 4 | 0.01 | 0.01 | 3 | 0.02 | 0.01 | 1 | 0.01 | 0.01 |
| 2006 | 2 | 0.01 | 0.01 | 2 | 0.01 | 0.01 | 0 | 0.00 | 0.00 |
| 2007 | 3 | 0.01 | 0.01 | 2 | 0.01 | 0.01 | 1 | 0.01 | 0.01 |
| 2008 | 3 | 0.01 | 0.01 | 2 | 0.01 | 0.01 | 1 | 0.01 | 0.01 |
| 2009 | 4 | 0.01 | 0.01 | 4 | 0.01 | 0.01 | 0 | 0.00 | 0.00 |
| 2010 | 8 | 0.01 | 0.01 | 7 | 0.02 | 0.01 | 1 | 0.00 | 0.00 |
| 2011 | 9 | 0.01 | 0.01 | 5 | 0.01 | 0.01 | 4 | 0.01 | 0.01 |
| 2012 | 4 | 0.00 | 0.00 | 2 | 0.00 | 0.00 | 3 | 0.01 | 0.01 |
| 2013 | 24 | 0.02 | 0.02 | 11 | 0.02 | 0.02 | 13 | 0.02 | 0.02 |
| 2014 | 19 | 0.01 | 0.01 | 7 | 0.01 | 0.01 | 12 | 0.02 | 0.02 |
| 2015 | 19 | 0.01 | 0.01 | 9 | 0.01 | 0.01 | 12 | 0.01 | 0.01 |
| 2016 | 19 | 0.01 | 0.01 | 15 | 0.02 | 0.02 | 2 | 0.00 | 0.00 |
| 2017 | 12 | 0.01 | 0.01 | 3 | 0.00 | 0.00 | 10 | 0.01 | 0.01 |
| 2018 | 27 | 0.01 | 0.01 | 8 | 0.01 | 0.01 | 18 | 0.01 | 0.01 |
| Total | 137 | 0.01 | 0.01 | 79 | 0.01 | 0.01 | 78 | 0.01 | 0.01 |
