# Supplementary material for: Trend of cancer mortality of the female reproductive system in China from 2005 to 2018 and prediction to 2035: A log-linear regression and Bayesian age-period- cohort analysis
Source: PLoS One. 2025 Aug 11;20(8):e0328869. doi: 10.1371/journal.pone.0328869 (PMC12338781; doi:10.1371/journal.pone.0328869)
Supplement: Attachment — Submitted filename: Response to Reviewers.doc [file pone.0328869.s004.doc]

PLOS ONE

April 2025

PONE-D-25-02080

Trend of cancer mortality of the female reproductive system in China from 2005 to 2018 and prediction to 2035: a Log-linear regression and Bayesian age-period- cohort analysis

Dear editor and reviewers,

We appreciate editor and reviewers very much for their positive and constructive comments and suggestions on our manuscript entitled “Trend of cancer mortality of the female reproductive system in China from 2005 to 2018 and prediction to 2035: a Log-linear regression and Bayesian age-period-cohort analysis” (Manuscript ID: PONE-D-25-02080). Those comments are all valuable and very helpful for revising and improving our paper, as well as the important guiding significance to our researches. Based on these comments and suggestions, we have made careful modification on the original manuscript.

On the separate pages, we provided our response to the comments and suggestions, point by point, and highlighted the changes in the marked copy of the revision. We hope that our revision will be approved by the experts and reviewed favorably.

Sincerely,

Weixia Nong, MD

Reviewer #1: Thank you for asking me to review the manuscript. For ease and compliance for editing, I had to convert and download as MSM instead of PDF.
The manuscript is well written and the information provided is useful to the scientific world. However I wish to observe and suggest as follows:
1. Title: The title may have to be edited as shown in the annotations in the attached suggestion for clarity.

**Response：**Thank you for your reasonable suggestions. But we didn't find the attachment. Could you please send it again.
2. Main text: Other errors and suggestions as attached in the annotations in the attachment.

**Response：**Thank you for your careful review. But we didn't find the attachment. Could you please send it again.
3. Discussion: The first paragraph here is essentially a repetition of the results, so should either be expunged or rephrased.

**Response：**Thank you for your reasonable suggestions. We have deleted.

Reviewer #2:

1.Review the manuscript for some typos.

**Response：**Thank you for your careful review. We have checked and modified.
2. Considering C51-58 as a whole - Since these involve topographies with different risk factors, incidence, and mortality, I suggest that the authors at least present a table with showing rates of each.

**Response：**Thank you for your reasonable advice. We have added the mortality rates of eight female reproductive system cancers in the Supplementary Material (Table S1-S8), and added description in the results: “It was further found that among the female reproductive system cancers, cervical uteri cancer had the highest ASMR (3.34 per 100,000) and placenta cancer had the lowest ASMR (0.01per 100,000) (Table S1-S8).”
3. For the first mortality rates presented in results, Are these crude or age-standardized rates?

**Response：**Thank you for your careful review. The first mortality rate that emerged in the results was the crude mortality rate, and the age standardized mortality rate was expressed by ASMR.
4. How was the ASMR calculated and which was the reference population? Describe in methods.

**Response：**Thank you for your reasonable suggestions. We have added in the methods section: “To eliminate the effect of the age structure of the population on the level of death, it is necessary to calculate the age-standardized mortality rate (ASMR), that is, the mortality rate calculated according to the age structure of a certain standard population. In this study, the standard population used was the population composition of the sixth national census in 2010 released by the National Bureau of Statistics of China(20).”
5. Describe in Methods how was the age groups achieved.

**Response：**Thank you for your reasonable suggestions. We have supplemented in the methods section: “The age groups in this study were divided into one age group at intervals of five years as stated in the Chinese Cancer Registry Annual Report.”
6. The authors perform a discussion of risk of individual female reproductive malignancies, but their research did not approach individual topographies. I suggest, some data of individual topographies be given.

**Response：**Thank you for your reasonable request. We have added the mortality rates of eight female reproductive system cancers in the Supplementary Material (Table S1-S8).
7. In limitations, it is said that this is a cross-sectional study. So, I suggest revising.

**Response：**Thank you for your reasonable suggestions. We have deleted.
