# Supplementary material for: Trend of cancer mortality of the female reproductive system in China from 2005 to 2018 and prediction to 2035: A log-linear regression and Bayesian age-period- cohort analysis
Source: PLoS One. 2025 Aug 11;20(8):e0328869. doi: 10.1371/journal.pone.0328869 (PMC12338781; doi:10.1371/journal.pone.0328869)
Supplement: Attachment — Submitted filename: Response_to_Reviewers_auresp_2.doc [file pone.0328869.s005.doc]

PLOS ONE

June 2025

PONE-D-25-02080

Trend of cancer mortality of the female reproductive system in China from 2005 to 2018 and prediction to 2035: a Log-linear regression and Bayesian age-period- cohort analysis

Dear editor and reviewers,

We appreciate editor and reviewers very much for their positive and constructive comments and suggestions on our manuscript entitled “Trend of cancer mortality of the female reproductive system in China from 2005 to 2018 and prediction to 2035: a Log-linear regression and Bayesian age-period-cohort analysis” (Manuscript ID: PONE-D-25-02080). Those comments are all valuable and very helpful for revising and improving our paper, as well as the important guiding significance to our researches. Based on these comments and suggestions, we have made careful modification on the original manuscript.

On the separate pages, we provided our response to the comments and suggestions, point by point, and highlighted the changes in the marked copy of the revision. We hope that our revision will be approved by the experts and reviewed favorably.

Sincerely,

Weixia Nong, MD

Required revisions:

The manuscript requires a complete revision of academic writing to enhance clarity, coherence, and overall readability. Please ensure the writing style aligns with scholarly standards.

**Response：**We reviewed and improved the full text again to make the expression of this article clearer and more in line with academic standards. Thank you very much to the editors and experts for their careful review and valuable comments.

1. **Discussion - Reference Citation:** In the second paragraph of the discussion, you mention "the 2024 edition of the Global Obesity Map released by the World Obesity Federation." Please provide a complete and proper reference citation for this source in your reference list.

**Response：**Thank you for your reasonable suggestions. We have supplemented the references.

2. **Discussion - Third Paragraph:** The sentence "These reasons may be due to the increasing trend in the mortality rate of the female reproductive system cancer in urban areas was more pronounced than in rural areas, leading to a narrowing of the mortality gap between urban and rural areas" requires revision for clarity and grammatical correctness. Please, also be mindful of potential ecological fallacy when making causal inferences from population-level data.

**Response：**Thank you for your reasonable suggestions. We strongly agree with this statement. We also recognize that such an explanation is inappropriate, so we have deleted it.

3. **Methodological Note:** Please acknowledge in your limitations section that the reference population used was China's population, which limits the generalizability of direct rate comparisons to other countries or global contexts.

**Response：**Thank you for your reasonable suggestions. We have added: “ Also, the fact that these data were obtained from the Chinese population limits the generalizability of direct rate comparisons with other countries or globally.”
